# Association between insurance status and mortality in individuals with albuminuria: an observational cohort study

**DOI:** 10.1186/s12882-016-0239-1

**Published:** 2016-03-09

**Authors:** Milda R. Saunders, Ana Catherine Ricardo, Jinsong Chen, Marshall H. Chin, James P. Lash

**Affiliations:** University of Chicago Medicine, 5841 S. Maryland, MC 5000, Chicago, IL 60637 USA; University of Illinois at Chicago, 820 Wood St, MC 793, Chicago, IL 60612 USA; University of Chicago Medicine, 5841 S. Maryland, MC 2007, Chicago, IL 60637 USA

**Keywords:** Albuminuria, Insurance, NHANES, Chronic kidney disease

## Abstract

**Background:**

In the general population, the association between uninsurance and mortality is well established. We sought to evaluate the association of health insurance status with mortality among working-age participants with albuminuria in the Third National Health and Nutrition Examination Survey, 1988–1994 (NHANES III).

**Methods:**

We used data from non-elderly adult participants (18–64) of NHANES III (1988–1994), a nationally representative study of the US civilian, noninstitutionalized population, who provided information on insurance and who had albuminuria, defined as a urine albumin-to-creatinine ratio [UACR] ≥ 30 mg/g and their subsequent mortality to December 31, 2006. Cox proportional hazards models were used to determine associations between insurance status and all-cause mortality and cardiovascular mortality in patients with CKD while adjusting in a stepwise fashion for sociodemographic factors, co-morbidities, and co-morbidity severity/control covariates.

**Results:**

In our sample of individuals with albuminuria (*n* = 903), mean estimated glomerular filtration rate (eGFR) was 101.6 ml/min/1.73 m^2^ with 4.7 % with an eGFR <60. Approximately 15 % of the sample was uninsured, 18 % had public insurance and 67 % had private insurance. Compared to individuals with private insurance, those with public insurance or no insurance were significantly more likely to be a racial or ethnic minority, to have income <200 % below the federal poverty level, to have less than high school education; and they were less likely to be married and to report good or excellent health, all *p* < 0.05. Being uninsured or having public insurance was associated with increased all-cause mortality in the fully adjusted model (HR 2.97 and 3.65, respectively, *p* < 0.05). There was no significant relationship between insurance status and cardiovascular mortality.

**Conclusions:**

In a nationally representative sample of individuals with albuminuria, uninsurance and public insurance were associated with increased mortality compared to the private insurance even after controlling for sociodemographic, health status, and health care variables. Improving access to care and the quality of care received may potentially reduce mortality in individuals with evidence of early CKD.

## Background

It is estimated that about 13 % of US working-age adults are currently uninsured [1]. The Affordable Care Act has the potential to continue to reduce the number of uninsured individuals in the US [2]. Health insurance is an important first step to health care access for these individuals. Uninsured adults receive less appropriate care and have worse outcomes compared to those with insurance [3–5]. More than 10 million adults in the US have albuminuria with preserved estimated glomerular filtration rate. Albuminuria is a well-established risk factor for chronic kidney disease (CKD) progression, cardiovascular events, and death [6–12]. Although lack of health insurance is known to be an important predictor of adverse outcomes among individuals with various medical conditions [3–5], its impact on mortality in adults with albuminuria has not been thoroughly evaluated. Prior work has shown that individuals who belong to racial and ethnic minority groups and those of lower socioeconomic status (SES) have a greater risk of albuminuria, and have greater health consequence associated with albuminuria [12–14]. The objective of this study is to evaluate the association of insurance status with all-cause and cardiovascular mortality among working-age adults with albuminuria in the Third National Health and Nutrition Examination Survey, 1988–1994 (NHANES III).

## Methods

### Study population

NHANES III, conducted by the National Center for Health Statistics (NCHS) between 1988 and 1994, is a nationally representative study based on a stratified, clustered, multistage probability sample survey of the civilian, noninstitutionalized population in the US. All participants provided informed consent. Participants underwent a home interview followed by an extensive physical examination and blood and urine sampling at a mobile examination center. Blood and urine samples and blood pressure were obtained during the physical examination. Self-reported information on sociodemographic characteristics and presence of medical conditions was collected during the home interview [15]. The survey protocol was approved by the NCHS institutional review board. Participants 65 years of age and older were excluded due to their low rates of uninsurance due to Medicare eligibility. Our sample included all NHANES III respondents aged 18–64 years with albuminuria who provided information on insurance.

**Fig. 1:**
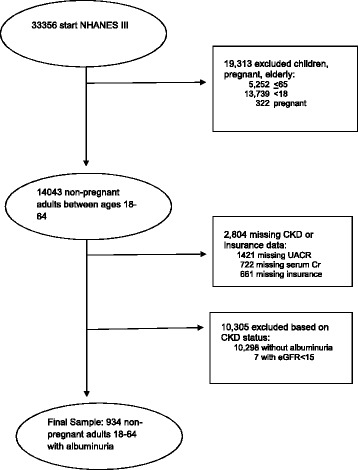
Study population and exclusions

Of the 33,356 NHANES III participants examined, we excluded 19,313 individuals who were pregnant, older than 65 or younger than 18 years. Of the 14,043, men and non-pregnant women between 18 and 65 years, we excluded 2804 individuals due to missing information on UACR, serum creatinine, or insurance status. We excluded 10,298 individuals without albuminuria and seven with eGFR < 15 ml/min/1.73 m^2^. Our final sample included 934 individuals with UACR ≥ 30 mg/g (Fig. 1). Compared to individuals in the final cohort, individuals who were excluded due to missing UACR were more likely to be older (50.3 vs 41.9 years, *p* < .001), and have an income <200 % federal poverty level (FPL) (51.2 vs 41.54 %, *p* = 0.03). Compared to individuals in the final cohort, individuals who were excluded because of missing insurance were more likely to be younger (35.2 vs 41.9 years, *p* < .001), be male (49.7 vs 37.4 %, *p* < .001), have < high school education (83.5 vs 73.8 %, *p* = 0.003), and to have an income <200 % FPL (73.3 vs 41.5 %, *p* < .001) (Data not shown).

### Definition of variables

Albuminuria was defined as a urine albumin-to-creatinine ratio [UACR] ≥ 30 mg/g. Estimated glomerular filtration rate (eGFR) was calculated using the 2012 CKD Epidemiology Collaboration creatinine equation [16]. We used the formula for correction of serum creatinine recommended in the NHANES III Data File Documentation [17]. Hypertension was defined as a BP >140 mmHg/>90 mmHg or the use of antihypertensive medications. Diabetes was defined as a history of diabetes, use of insulin or other medication to treat diabetes, a fasting blood glucose level ≥126 mg/dl, or a random blood glucose level ≥200 mg/dl. Hypercholesterolemia was a total cholesterol concentration ≥200 mg/dL. History of cardiovascular disease was defined by an affirmative answer to at least one of the following: history of heart attack, congestive heart failure, or stroke. Insurance status was obtained by patient self-report. At the time of interview patients were asked if they were covered by Medicare, Medicaid, CHAMPUS/VA/military insurance or private insurance in the last month. The answers were categorized as uninsured (none of the above), public insurance (Medicare or Medicaid), or private (all other) insurance.

### Outcome ascertainment

#### Mortality follow-up

Vital status was established using the NHANES III Linked Mortality File which provided follow-up for mortality through December 31, 2006. Probabilistic matching was used to link NHANES III participants with the National Death Index (NDI) to ascertain vital status. Matching was based on 12 identifiers for each participant (e.g., Social Security number, sex, and date of birth). Participants who were not matched with any death records were considered to be alive through the follow-up period. Cause of death was assigned by the NCHS based on the International Classification of Diseases, 10th Revision [18]. For this study, cardiovascular mortality was defined as death due to diseases of the heart, essential hypertension and hypertensive kidney disease, cerebrovascular disease, atherosclerosis, and other diseases/disorders of the circulatory system (codes 100–199) [19].

#### Statistical analysis

NCHS recommendations were followed to account for stratification and clustering in the survey design, as well as oversampling of ethnic minorities and elderly persons [17]. Continuous variables were expressed as weighted means (standard error) and categorical variables as weighted percentage. Baseline characteristics were compared across insurance status using analysis of variance for continuous variables and chi square for categorical variables.

Weighted age-adjusted all-cause and cardiovascular mortality rates were calculated by 1000 person/year. Cox proportional hazards models were used to determine the association of insurance status with all-cause and cardiovascular mortality in patients with albuminuria adjusting for important covariates in a hierarchical fashion: 1) Model 1: *sociodemographic factors* (age, gender, race/ethnicity, income, education, and marital status); 2) Model 2: Variables included in Model 1 plus *co-morbidities* (diabetes mellitus status, cardiovascular disease, smoking, body mass index, and cancer); 3) Model 3: Variables included in Model 2 plus *co-morbidity severity* (eGFR, systolic blood pressure, serum cholesterol, and hemoglobin A1C); and 4) Model 4: Variables included in Model 3 plus *co-morbidity management (*use of statin, use of ACEi). In addition, we evaluated for the presence of interaction between insurance status and age, gender, and race/ethnicity on all-cause mortality by adding interaction terms in the fully adjusted models. The proportional hazards assumption of the Cox models was examined using Schoenfeld residuals.

## Results

In our study population (Table 1), the mean age at the time of the interview was 42 years, 63 % of participants were women, 67 % were non-Hispanic White, 16 % were non-Hispanic Black, 6 % were Mexican American and 11 % were of other racial/ethnic background. In addition, approximately 37 % had income less than 200 % FPL, 74 % had less than high school education and 64 % were married. In this sample of NHANES participants, 40 % of participants reported excellent or very good health, 36 % had hypertension, 24 % had diabetes, 44 % had hypercholesterolemia, and 6 % reported cardiovascular disease. The mean eGFR was 102 mL/min/1.73 m^2^ and median UACR was 71 mg/g. Overall, 4.7 % of the participants had eGFR < 60 ml/min/1.73 m^2^ and 11.5 % had UACR ≥ 300 mg/g.

**Table 1:** Characteristics of individuals with albuminuria (age < 65) overall and stratified by insurance status

Covariates	Overall	Uninsured	Public insurance	Private insurance	p
N=	903	197	203	503	
Age	41.90 (0.69)	38.26 (1.77)	44.88 (1.93)	41.90 (0.81)	0.04
Age group					
< 45	57.39	67.65	44.82	58.56	0.045
45–65	43.54	35.87	55.54	42.05	0.079
Gender					
Male	37.38	31.02	36.53	39.00	0.541
Female	62.62	68.98	63.47	61.00	
Race					
Non-Hispanic Whites	66.67	51.03	49.86	74.55	<.001
Non-Hispanic Black	16.29	15.37	29.56	12.97	<.001
Mexican American	5.90	16.36	5.09	3.82	<.001
Other	11.14	17.24	15.49	8.65	0.108
Income					
< 100 FPL	17.54	41.76	40.55	7.27	<.0001
101–200 FPL	19.92	28.27	30.27	15.85	0.0014
> 200 FBL	62.54	29.97	29.18	76.88	<.001
FPL (Median, IQR)	193.8 (104.2,331.3)	96.6 (58.1,156.4)	95.6 (63.25,165.15)	250.6 (154.8,379.9)	
Education					
< High School	73.79	90.17	87.93	66.45	<.001
> = High School	26.21	9.83	12.07	33.55	
Marital Status					
Married	64.10	58.98	42.90	70.84	<.001
Previously Married	16.70	18.04	32.50	12.23	<.001
Never Married	19.19	22.98	24.61	16.93	0.200
Self-rated Health					
Excellent/Very Good	40.45	28.74	25.65	46.93	0.004
Good	37.42	36.83	33.16	38.68	0.595
Fair/Poor	22.13	34.43	41.20	14.39	<.001
Usual Source of Care (Yes)	83.02	65.93	88.39	85.35	<.001
Usual Care Provider (Yes)	72.95	50.58	70.03	78.64	<.001
Smoking (Yes)	33.75	29.77	45.98	31.39	0.032
Hypertension (Yes)	36.14	34.12	45.22	34.18	0.248
Systolic BP, mm Hg	127.31 (0.96)	127.43 (3.08)	128.08 (3.03)	127.09 (1.28)	0.921
Diastolic BP, mm Hg	77.80 (0.55)	78.34 (1.44)	78.19 (1.34)	77.57 (0.74)	0.670
BMI					
< 30 kg/m2	65.28	62.77	62.57	66.55	0.801
> =30 kg/m2	34.72	37.23	37.43	33.45	
Diabetes (Yes)	23.60	25.44	36.05	19.91	0.010
HbA1C	6.02 (0.10)	6.16 (0.18)	6.21 (0.18)	5.94 (0.14)	0.240
History of CVD (Yes)	5.53	5.23	12.39	3.79	0.010
ACE Use (Yes)	13.19	20.88	18.72	10.62	0.097
Cholesterol (Yes)	43.80	49.71	40.23	43.45	0.427
Statin Use (Yes)	4.48	10.33	7.52	2.81	0.277
Mean eGFR	101.61 (1.18)	105.40 (3.20)	97.87 (4.02)	101.76 (1.48)	0.250
eGFR < 60 (%)	4.74	3.46	9.30	3.81	0.177
Albuminuria Median (IQR)	70.92 (43.02,154.42)	69.48 (40.47,123.40)	98.28 (46.51,265.43)	67.09 (42.46,143.50)	
UACR					
30–299	88.52	90.99	83.47	89.32	0.288
> =300	11.48	9.01	16.53	10.68	

Note: History of CVD is defined as has history of heart failure or stroke

The weighted prevalence of uninsured, public insurance and private insurance was 15, 18 and 67 %, respectively. Compared to individuals with private insurance, those with public insurance or no insurance were significantly more likely to belong to a racial/ethnic minority group, to have income <200 % FPL, and to have less than high school education (*p* < 0.05 for each comparison). Uninsured individuals were younger and less likely to report a usual source of care than their insured counterparts (private or public), all *p* < 0.05. Individuals with public insurance differed from their privately (and non-insured counterparts) in that they were more likely to be non-Hispanic Black, to report fair/poor health, to be previously married, and to have hypertension, diabetes or cardiovascular disease, all *p* < 0.05. There were no significant differences in mean eGFR, UACR, statin use, or ACE-i use by insurance status.

### All-cause and cardiovascular mortality by insurance status and access to care for individuals with albuminuria

The age-adjusted all-cause mortality rate for individuals with albuminuria was 13.5/1000 person-year (Table 2). Crude rates of all-cause mortality were higher in in the uninsured and individuals with public insurance than in those with private insurance (17.8 and 24.1 vs 10.4, respectively). A similar pattern was observed with cardiovascular mortality rates.

**Table 2:** Mortality rate by insurance status for individuals with albuminuria

Variable (*N* = 915)	Age-adjusted mortality rate (All-cause) per 1,000 person-year	Age-adjusted mortality rate (CV) per 1,000 person-year
Overall	13.53	5.16
Insurance status (ref: Private)	10.39	4.14
Uninsured	17.76	7.02
Public	24.06	8.06

In fully adjusted models, compared to individuals with private insurance, being uninsured was associated with increased risk for all-cause mortality (HR 2.97, 95 % CI 1.29–6.85). Compared to individuals with private insurance, having public insurance was also associated with increased mortality (HR 3.65, 95 % CI 1.74–7.67) (Table 3). There was a suggestion of effect modification by eGFR; however, due to low proportion of individuals with eGFR < 60 stratified analyses could not be done. We found no interaction between insurance status and race/ethnicity, gender, or age.

**Table 3:** Risk for all-cause mortality by insurance status and access to care for individuals with albuminuria

	All-cause mortality	P	CV mortality	P
N=	913		913	
Unadjusted HR				
No Insurance V Private (REF)	1.30(0.77,2.21)	0.316	1.30(0.50,3.40)	0.581
Public V Private	2.75(1.61,4.68)	<.001	2.30(1.12,4.72)	0.024
HR, Model 1^a^				
No Insurance V Private (REF)	1.80(0.74,4.34)	0.189	1.96(0.66,5.77)	0.219
Public V Private	1.70(0.87,3.34)	0.118	1.73(0.63,4.76)	0.285
HR, Model 2^b^				
No Insurance V Private (REF)	1.86(0.71,4.87)	0.199	1.88(0.46,7.58)	0.370
Public V Private	1.66(0.81,3.43)	0.165	1.48(0.47,4.65)	0.492
HR, Model 3^c^				
No Insurance V Private (REF)	1.90(0.83,4.34)	0.125	1.95(0.56,6.85)	0.288
Public V Private	1.87(1.01,3.46)	0.046	1.57(0.52,4.73)	0.417
HR, Model 4^d^				
No Insurance V Private (REF)	2.97(1.29,6.85)	0.012	2.15(0.44,10.46)	0.337
Public V Private	3.65(1.74,7.67)	<.001	3.21(0.77,13.43)	0.107

^a^Model 1: Sociodemographic: age, gender, race/ethnicity, PIR (poverty income ratio), education, marital status

^b^Model 2: Model 1+ Diabetes (Y/N), Cardiovascular disease (Y/N), smoking (Y/N), Body mass index, cancer (y/n)

^c^Model 3: Model 2+ estimated glomerular filtration rate (eGFR), systolic blood pressure, cholesterol (<200 mg/dL, >200 mg/dL HgbA1c (<7, >7)

^d^Model 4: Model 3 + statin(Y/N), angiotension converting enzyme (ACE)-Inhibitor (yes/no)

The age-adjusted cardiovascular mortality rate for individuals with albuminuria was 5.16/1000 person-year (Table 2). In fully adjusted models, the cardiovascular mortality risk was similar between individuals with private insurance vs uninsured vs public insurance. We found no significant interaction between insurance status and age, gender, race, or eGFR on cardiovascular mortality.

## Discussion

In this nationally representative sample of individuals with albuminuria and preserved eGFR, we found that both uninsurance and public insurance were associated with a significantly higher risk for mortality compared to private insurance. Uninsured individuals with albuminuria had nearly a three-fold higher risk for all-cause mortality than their privately insured counterparts after controlling for socioeconomic and clinical characteristic. Similar to our findings, the Kidney Early Evaluation Program (KEEP), a community-based health screening program, found an increased risk for death in uninsured participants with CKD stages 1–2 as compared to those with private insurance [20]. Our study provides corroborative evidence in a sample representative of the US population.

We found that in a working age, US population with albuminuria, 15 % were uninsured, 18 % had public insurance, and 67 % had private insurance. Uninsurance was higher than a previous examination of uninsured adults with CKD because we looked only at individuals younger than 65 whom comprise over 90 % of the uninsured with CKD [21]. Our uninsurance rate was closer to Wilper et al. who also used NHANES to examine uninsurance in working age adults [5, 22].

In the general population, the association of uninsurance with increased risk for mortality is well established [5, 23, 24]. However, the relationship between insurance status and outcomes has not been as well studied in the CKD population. In a cross-sectional analysis of data from NHANES, uninsured individuals with CKD were less likely to have controlled hypertension or to receive an angiotensin-converting enzyme inhibitor (ACEi) than those with insurance [21, 25]. At the time of our study, rates of both ACEi and statin use were low in the general population [26, 27]. Although we did not find a significant difference in rates of ACEi use across insurance categories, adding ACEi and statin to our final model increased both the magnitude and significance of the association between uninsurance and mortality. These findings suggest that ACE/statin are important mediators of the association or that they are potentially in the causal pathway.

Publicly insured individuals with albuminuria also had a significantly higher all-cause mortality rate than their privately insured counterparts. This finding was true both in the crude model and after adjusting for socioeconomic and clinical characteristics. For most working-age adults, eligibility for public insurance is dependent on income and poor health [28]. In a national survey data study, Sorlie et al. also reported increased mortality in individuals with public insurance as compared to those with employer provided insurance [29]. Sorlie also found that in certain groups, public insurance was associated with increased mortality compared to uninsurance [29].

Our findings that health insurance is an important predictor of mortality in individuals with kidney disease, many of whom had early signs of kidney disease, has important implications in the current climate of healthcare reform. The number of uninsured in the US has decreased due both to the economic recovery and implementation of Affordable Care Act. The uninsurance rate for working age adults, 18–64 years old, was estimated to be 12.4 % in late 2014 [1]. Due to targeted policies, poor working adults saw the largest gains in insurance coverage, particularly in Medicaid expansion states [1]. However, the uninsured have gained coverage through a combination of expansion of employer-based coverage, health insurance exchanges, extended parental coverage, and Medicaid expansion [30]. More individuals may potentially benefit given the current eligibility, and requirement, for nearly all working age adults to obtain insurance. In addition, the association of public insurance with increased mortality will likely be reduced as eligibility is based on income rather than health status.

We found a less convincing relationship between insurance status and cardiovascular mortality. The uninsured did not have a significantly different CV mortality than their privately insured counterparts. This finding seems surprising given the importance of medical care in managing cardiovascular risk factors and intervening on cardiovascular events. One possible explanation is that uninsured individuals with CKD and CV disease may remain uninsured for less time than their healthier counterparts. Due either to the number or severity of CV risk factors, they are more likely to meet criteria for public insurance. One final explanation is that since everyone regardless of insurance status is eligible for medical care during a cardiovascular emergency, the effect of insurance status on access to life-saving CV care is less important.

Our study had several limitations. Because of the design of NHANES, we only had access to a single UACR and eGFR determination, rather than the multiple measures. However, prior studies have used this method to ascertain CKD [21, 26]. In addition, we were able to determine insurance status only at a single point in time. Patients may have had only a limited period of uninsurance or insured patients may have subsequently lost their insurance. Nonetheless, our classification of insurance status based on a single assessment was associated with significant differences in long-term outcomes.

## Conclusions

In conclusion, in a nationally representative sample of individuals with albuminuria and preserved eGFR, both uninsurance and public insurance were associated with increased mortality compared to private insurance, even after controlling for sociodemographic, health status, and health care variables. Given the burdens of kidney disease both to the affected individuals, families, and the health care system, we must wait to see if efforts to provide comprehensive health care—by improving access to care and the quality of care received—will be able to help to reduce costs, morbidity and mortality for those with early evidence of CKD.

### Availability of data and materials

The datasets supporting the conclusions of this article are available in the National Center for Health Statistics data repository. Third National Health and Nutrition Examination Survey (NHANES III) data is available at doi.org/10.3886/ICPSR02231.v1 and http://www.cdc.gov/nchs/nhanes/nhanes3/data_files.htm## with linkage to the National Death Index at http://www.cdc.gov/nchs/data_access/data_linkage/mortality.htm.

## Abbreviations

ACEi: angiotension converting enzyme inhibitor
BP: blood pressure
CI: confidence interval
CKD: chronic kidney disease
CV: cardiovascular
eGFR: estimated filtration rate
FPL: federal poverty level
NCHS: National Center for Health Statistics
NHANES: National Health and Nutrition Examination Study
UACR: urine albumin-to-creatinine ratio
US: United States
